# Effect of Multiple Factors on Foam Stability in Foam Sclerotherapy

**DOI:** 10.1038/s41598-018-33992-w

**Published:** 2018-10-24

**Authors:** Taoping Bai, Wentao Jiang, Yu Chen, Fei Yan, Zhi Xu, Yubo Fan

**Affiliations:** 1 Laboratory of Biomechanical Engineering, Department of Applied Mechanics, Sichuan University, Chengdu, 610065 China; 2 Department of Biomedical Engineering, The Hong Kong Polytechnic University, Hong Kong, China; 3 Institute for Disaster Management and Reconstruction (IDMR), Sichuan University — The Hong Kong Polytechnic University, Chengdu, 610065 China; 4 School of Biological Science and Medical Engineering, Beihang University, Beijing, 100191 China

## Abstract

Foam sclerotherapy is a widely used treatment for varicose veins. However, complications caused by poor foam stability still remain. Most studies ignore multiple influencing factors and only study a single factor. Furthermore, a stable foam preparation using different preparation conditions has not been developed. This study aimed to explore the changing laws of foam stability under multifactorial conditions, and to determine the influence of various factors and optimal preparation conditions on the half-life of foam. A two-level orthogonal test was conducted using four factors (syringe size, surfactant, preparation temperature, and pump speed). Classifications were established as follows: syringe sizes, 2.5 mL and 5 mL; surfactant concentrations, 6% and 0%; preparation temperatures, 20 °C and 10 °C; and pump speeds, 250 mm/s and 125 mm/s, respectively. Eight experimental group (EG) multi-factor combinations were tested. Half-life and drainage time were recorded for analysis. The initial drainage time was within 200 s, but the difference between the groups was also about 200 s. The drainage rate curves of all EGs gradually increased over time. Conversely, the foam half-life extended by about 10 times for the four factors. In addition, the analyses revealed that the order of influence was surfactant >temperature >pump speed >syringe size. The most stable foam preparation was determined. Syringe size, surfactant, temperature, and pump speed had markedly observable influences on foam half-life. A combination of multiple factors can be used to prepare a more stable foam in clinical scenarios and to suitably superimpose favorable conditions to avoid unfavorable conditions.

## Introduction

Varicose veins are high-incidence venous diseases^1,2^. Foam sclerotherapy is being gradually accepted as a treatment modality for varicose veins, as it is a simple, minimally invasive operation with good efficacy. The sclerosing drug in a liquid form is mixed with a gas to produce foam which is immediately injected into the varicose vein. The sclerosant foam occludes the varicose veins for therapeutic purposes^3^. In addition, the safety of this therapy for elderly patients has been established^4^. Foam sclerosing agents are also used in other tissue closure procedures^5^.

However, foam sclerotherapy can lead to complications such as pulmonary embolism, deep vein thrombosis, phlebitis, visual disorders, and stroke^6–8^. A major reason is poor foam stability, which causes the decaying foam to be easily diluted, allowing dissemination through the body. These limitations have attracted the attention of several researchers. Rial and Ceulen studied the effects of the concentrations of gases and drugs on foam stability^9–12^. Previous studies reported that the optimal pump speed of Tessari’s method was highly advantageous for foam stability^13^. A smaller syringe size and a lower temperature are factors that contribute to greater stability of the foam^14,15^. Furthermore, the addition of certain surfactants can also enhance foam stability^16^. However, to the best of our knowledge, studies examining the effect of multiple factors on foam stability are lacking. The degree of influence of each of these factors on foam stability has not been evaluated, and stable foam preparation using different preparation conditions has not been accomplished. As a result, therapeutic strategies based on the current research on sclerosant foam stability is less applied in clinical settings. This has directly hindered the development and application of foam sclerotherapy and foam improvement. A combination of these factors can provide clinically important information regarding foam decay under the influence of various conditions. Concurrently, we propose a multi-factor correlation judgment equation to provide theoretical support for multi-factor research.

## Materials and Methods

**Figure 1:**
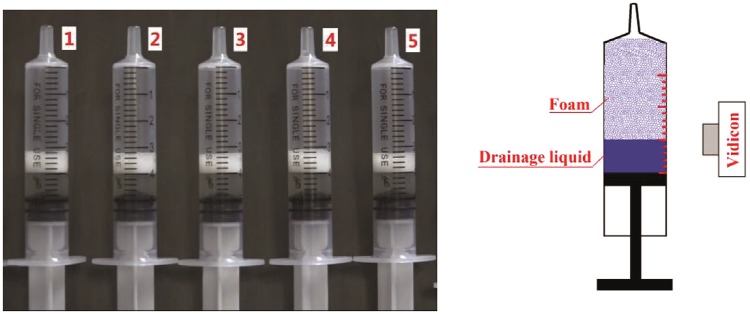
Data records of experimental.

Sodium morrhuate (0.1 g in 2 mL injection) was chosen as the experimental drug due to its good stability. The gas used was CO_2_, and the liquid to gas ratio was 1:4. Tessari’s method was used to prepare the foam. This method uses two syringes of the same type; one syringe contains one part of the drug and the other contains four parts of CO_2_. The syringes are then connected to a medical three-way valve at an angle of 90° with each other. After 10 rounds of push and pull, the three-way switch is turned off. Then, the syringes are pushed 10 times to complete the foam preparation. The prepared foam is immediately removed and placed on a horizontal desktop. In our study, this whole process was video-recorded for later observation (Fig. 1). The stability of the foam was quantitatively analyzed using stability parameters.

### Determination of optimal pushing speed of the 2.5 mL syringe during foam preparation

**Figure 2:**
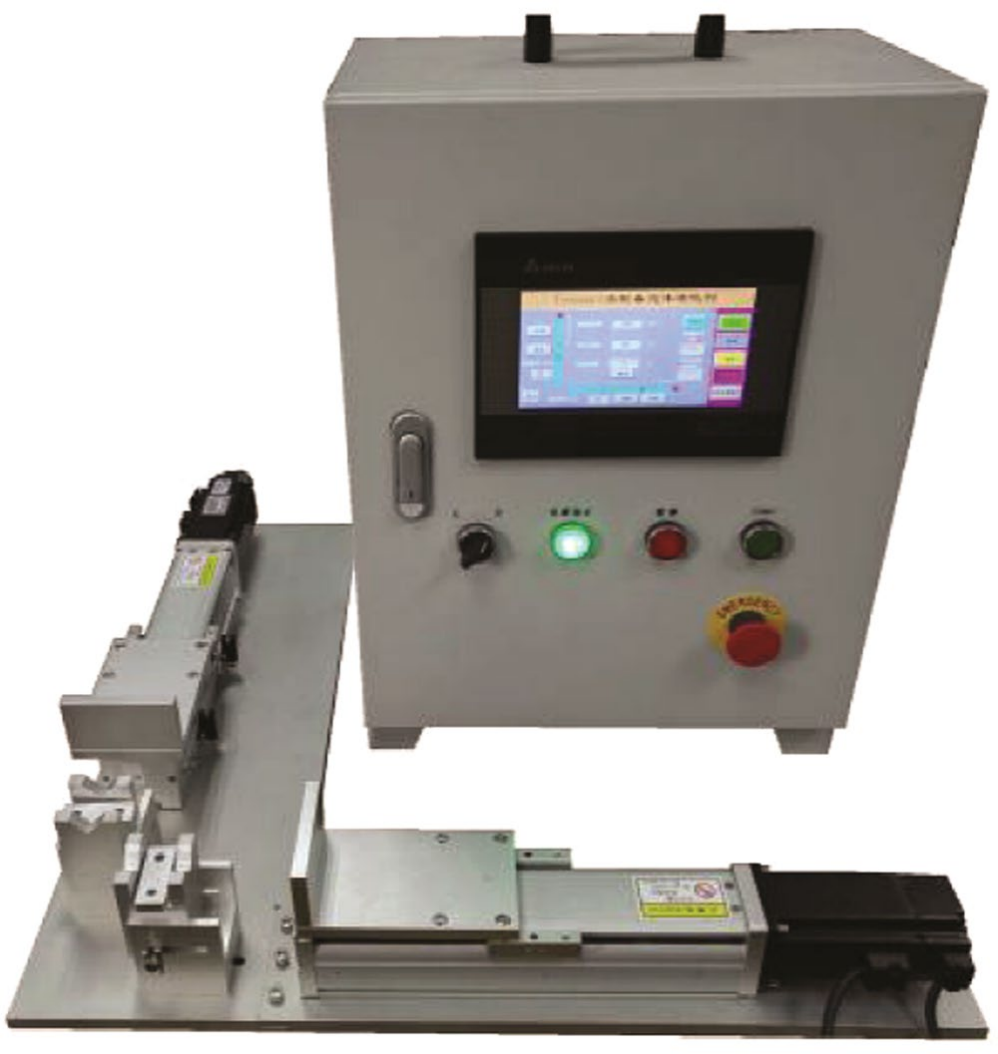
Sclerosing foam preparation machine.

To ensure a constant pump speed and cycling rate, we used a laboratory-made automatic device for preparing the sclerosing foams (Fig. 2)^17^. When comparing the average speed of the device during actual operation with the set one, the maximum error was only 2.95%^17^. This confirmed that the device met the research needs and was suitable for foam preparation using the Tessari method under the specified speed conditions.

In the experiment, 2.5 mL syringes (Jiangxi Hongda Medical Equipment Group Co., Ltd.) were used to prepare the foam in the preparation machine. The optimal injection speed required was determined separately. Therefore, eight pump speeds (100, 200, 250, 300, 325, 350, 375, and 400 mm/s) were selected for the stability experiments to determine the best pump speed for 2.5 mL syringes.

**Figure 3:**
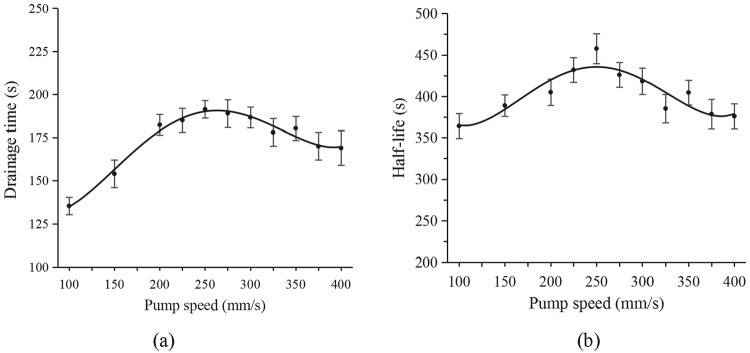
Drainage time (**a**) and half-life (**b**) curves from the 2.5 mL pump speed experiment.

The experiments with the 2.5 mL syringes showed that the drainage time and half-life changed with the pump speed used (Fig. 3a,b, respectively). The drainage time curve showed that the range was between 100 s and 200 s. When the pump speed was 100 mm/s, the drainage time was the shortest. When the speed was higher, the drainage time initially increased and eventually decreased. The longest drainage time (about 190 s) was at a speed of 250 mm/s. The half-life curve shows that the variation range was between 350 s and 475 s. The maximum half-life was approximately 450 s, which was also observed when the injection rate was 250 mm/s. This phenomenon was similar to the results of a previous study^13^. Therefore, the optimal pump speed of the 2.5 mL syringe was 250 mm/s, and a pump speed of 125 mm/s was chosen as the control speed.

### Surfactant selection

The foaming properties of five excipients (propanediol, Tween 80, macrogol 4000, lecithin, and poloxamer 188) were tested at different concentrations. The experimental methods and groups are described in detail in our previous paper^18^. The experimental results of surfactant influence show that the addition of the surfactant can significantly enhance the stability of the foam. Different surfactants show a significant difference with respect to foam stabilization. Among them, the effect of poloxamer 188 was the most apparent; a high-stability foam can be obtained at room temperature with poloxamer 188. Moreover, the half-life of the foam was enhanced ten-fold. In the experimental concentration range, the longest half-life had a 16-fold increase. Based on the previous experimental results, the surfactant selected in this experiment was poloxamer 188. Additionally, in accordance with drug safety guidelines for humans, 6% g/mL of poloxamer 188 was used.

### Overall experimental design

To investigate the multifactorial effects, the syringe size, surfactant, preparation temperature, and pump speed were considered for orthogonal experiments. The smaller the syringe size, the more stable was the foam under the same conditions; hence, 2.5 mL and 5 mL syringes were chosen. The clinical preparation of the foam was completed at a temperature of 20 °C, and 10 °C was chosen as the control temperature. The four-factor and two-level orthogonal experimental group (EG) designs are shown in Tables 1,2, respectively.

**Table 1:** Groups of multi-factor experiments.

Classification	Syringe Size A	Surfactant B	Prepare Temperature C	Pump Speed D
1	5 ml	0%	10 °C	125 mm/s
2	2.5 ml	6%	20 °C	250 mm/s

**Table 2:** L_8_ (2^4^) Orthogonal experiment table.

Grouping Factors	Syringe Size	Surfactant	Prepare Temperature	Pump Speed
EG 1	1	1	1	1
EG 2	1	1	1	2
EG 3	1	2	2	1
EG 4	1	2	2	2
EG 5	2	1	2	1
EG 6	2	1	2	2
EG 7	2	2	1	1
EG 8	2	2	1	2

The samples for the various EGs were simultaneously fabricated under the same conditions; none of the foam preparation parameters was altered. The number of pump cycles and pump speed also remained fixed, and each set of experiments was repeated five times. All the experimental apparatuses and drugs were maintained at 25 °C for 30 min before the start of the experiments, and all the experiments were performed at approximately 1,650 ft. above sea level. In addition, the height of the camera and its focal length were carefully adjusted. The recorded data included: (1) drainage rate, defined as drainage water divided by drug volume, and duration of foam drainage; and (2) foam half-life (***T***), defined as the time at which the foam drainage rate was 50%. Half-life is a key factor in describing the foam decay process and is also representative of foam stability.

## Results

**Figure 4:**
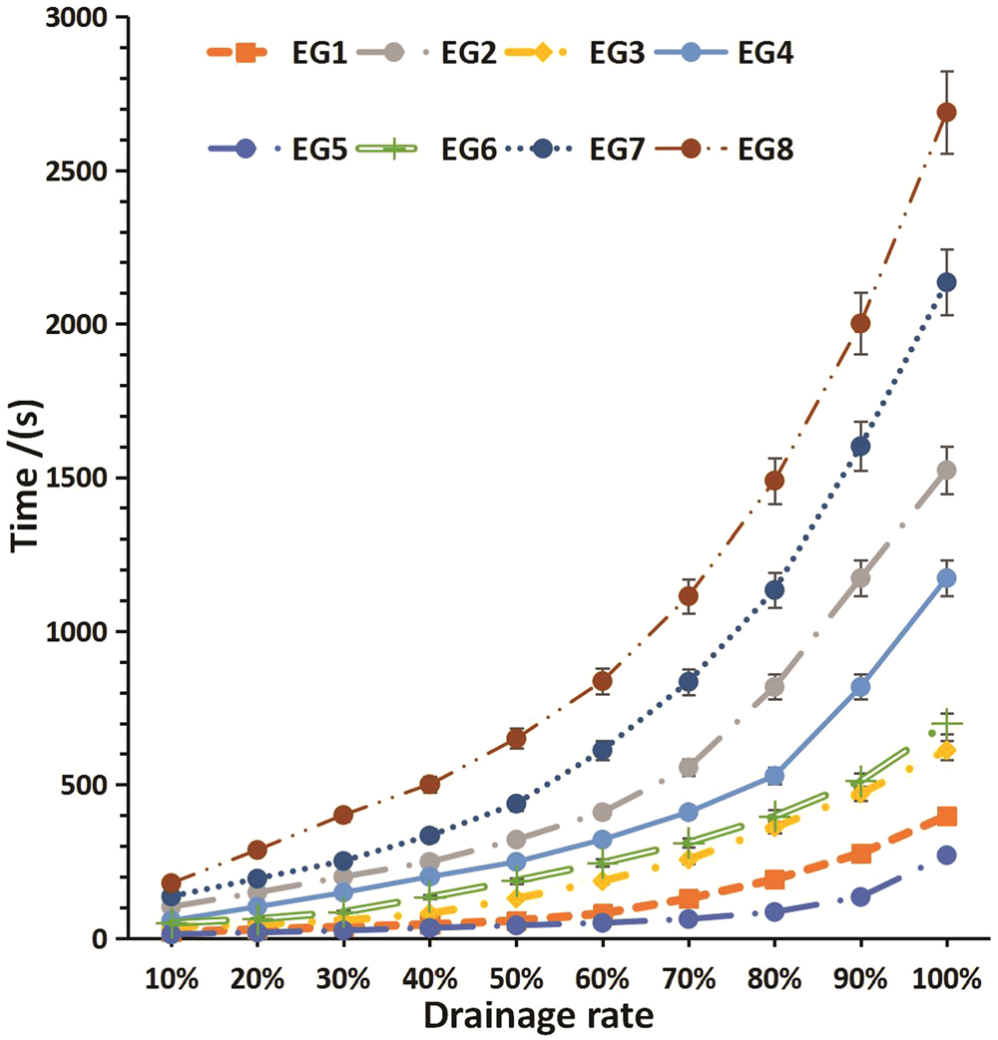
Overall drainage rate curve by experimental group.

The overall drainage curve of the multivariate experiment is shown in Fig. 4, with EG5 having the lowest drainage curve and a total water withdrawal time within 250 s. EG8, EG7, and EG2 had a total water withdrawal time of up to approximately 2,700 s, 2,200 s, and 1,500 s, respectively. The overall initial drainage time was within 200 s; however, the differences between the groups were also approximately 200 s. The rate curves of all EGs gradually increased over time, and the rate of growth was markedly observable.

**Figure 5:**
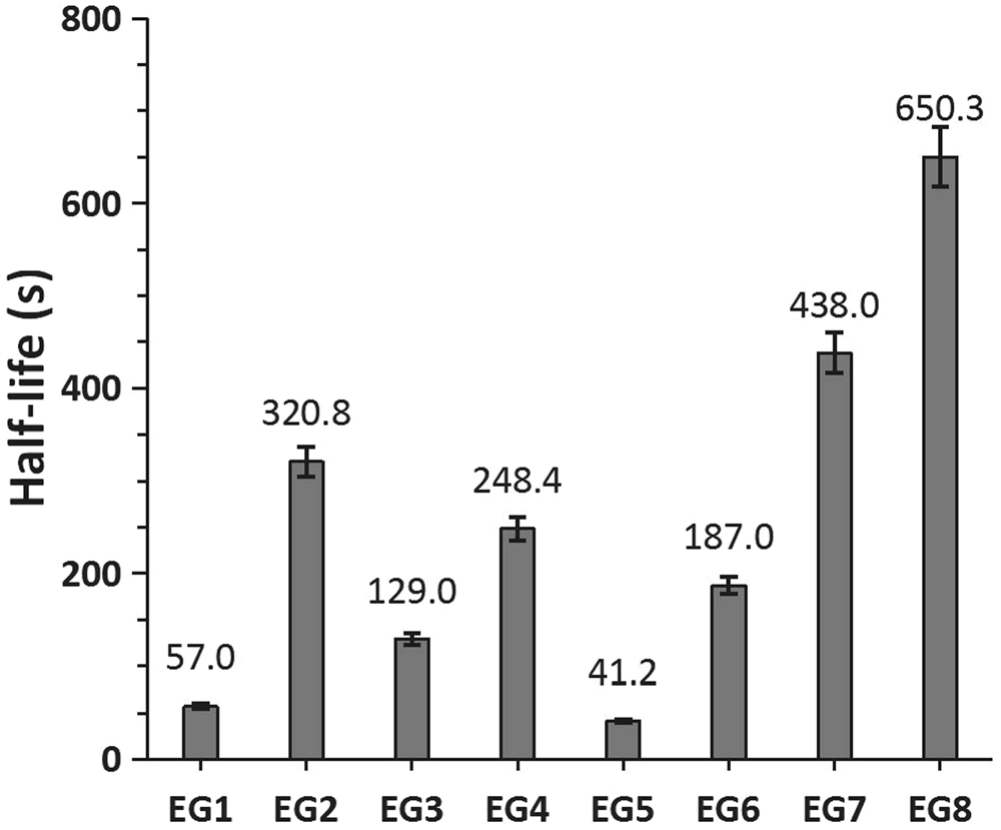
Half-life histogram by experimental groups.

The half-life histogram for the multivariate orthogonal experiment is shown in Fig. 5. EG5 had the least half-life (41.2 s), whereas EG8 had the highest half-life (650.3 s), which was approximately 600 s longer than that recorded for EG5.

Figure 3 confirms the influence of pump speed on foam stability, suggesting that the optimal injection speed was 250 mm/s when a 2.5 mL syringe was used for preparing the foam at 20 °C. Moreover, EG2, EG4, EG7, and EG8 foams were more stable than the EG5 foam, and the stability of the foam greatly improved. EG8 yielded a 14-fold improvement in foam stability relative to EG5.

The results from this experimental design for the multifactorial analysis are shown in Table 3. The order of influence for the four factors was: surfactant >temperature >pump speed >syringe size. The best combination of the four factors and two levels was B_2_C_1_D_2_A_2_., that is, the most stable foam was obtained when the syringe size was 2.5 mL, the appropriate surfactant was added, the preparation temperature was 10 °C, and the injection speed was 250 mm/s. Table 3 shows that the synergistic effect of surfactants with other parameters was remarkable.

**Table 3:** Orthogonal Experimental Results Analysis Table.

	Syringe Size	Surfactant	Prepare Temperature	Pump Speed
${K}_{1}$	755.20	605.20	1466.05	665.20
${K}_{2}$	1316.45	1465.65	605.60	1406.45
$\overline{{K}_{1}}$	188.80	151.30	366.51	166.30
$\overline{{K}_{2}}$	329.11	366.41	151.40	351.61
R	140.31	215.11	215.11	185.31
Order	B > C > D > A			
Excellent level	A_2_	B_2_	C_1_	D_2_
Excellent combination	B_2_C_1_D_2_A_2_			

EG8 comprised adjusted preparation temperature, syringe size, pump speed, and surfactant, which extended the half-life of sodium morrhuate foams to approximately 10 min. During clinical treatment with foam sclerotherapy, the occlusion time of the blood vessel is approximately 10 min. Therefore, the foam prepared using EG8 conditions was the ideal preparation and can be applied in clinical settings.

## Discussion

In this study, multifactorial experiments incorporating various syringe sizes, surfactants, preparation temperatures, and pump speeds were conducted, and some valuable laws were identified. The potential side effects of the deposition of the foam and liquid at other parts of the body cannot be underestimated. Therefore, the unavailability of a method for preparing a uniform and stable foam has been a deterrent for the clinical use of foam sclerotherapy. Many improvements have occurred with respect to the conditions for foam preparation, but there has been no comprehensive research regarding the multiple factors involved in the preparation. The current available studies do not discuss foam preparation involving multiple factors; therefore, the clinical effect cannot be properly assessed, and this has hampered the development of sclerotherapy.

The energy of the foam is high; hence, the foam is very unstable. The effects of multiple parameters on foam stability are demonstrated in the difference in foam preparation and the different environments in which the decay occurs. The stability of the foam is determined by foam decay, which is demonstrated by gas diffusion and gravity induced inflow. The pressure differences and surface tension between the bubbles are the main causes of decay. The pressure inside the small foam is high and will spread to the big bubble. The uniformity of the foam directly affects the rate of diffusion between the bubbles. The viscosity and surface tension of the liquid may induce a change in the inflow velocity.

Small syringe sizes will result in more uniform foam, when pushed back and forth^14^. Therefore, gas diffusion slows down. This only changes the foam decay for a few seconds. Further, the impact of the syringe size is small (Table 3). For parameter B, surfactants can markedly change the surface tension of the solution, and the surface tension affects the pressure differences between the bubbles, thereby, changing the gas diffusion rate from beginning to end. Figure 5 and Table 3 also show that the addition of a surfactant can greatly affect the foam stability. Hence, focus should be given to the addition of surfactants and other optimal preparation conditions to provide a stable foam for clinical use. However, surfactants generally have a critical micellar concentration and cannot be used at high concentrations. Surfactants have been widely used in the manufacture of industrial foams. The poloxamer 188 in this study has been shown to be feasible for intravenous injection, but animal experimentation must be done to ensure further applications.

As parameter C, preparation temperature is the temperature at which the foam is formed and can affect the surface tension of the foam. At the same time, the temperature can change the viscosity of the liquid, causing the internal flow rate to change. It has a greater impact on the decay process (Fig. 5 and Table 3). Therefore, the effect of temperature on foam stability is significant. The faster the pushing speed (parameter D), the more uniform the foam, and the smaller the diameter, the lower the rate of foam diffusion. However, as the speed increases, the solubility of carbon dioxide increases. This causes a decrease in the amount of gas, an increase in the humidity of the formed foam, and an increase in the internal flow. The curve in Fig. 3 illustrates the effect of the entire pushing speed. Therefore, there is a need to determine the optimal speed for foam formation.

The overall result of multiple parameters (Fig. 5) is EG8, which increases the half-life by more than 10 times. The enhancement effect on stability is obvious. However, the value of EG8 is not a superposition of the individual effects of each parameter. It is possible that their superposition will weaken to some extent, which may be because the changes in the internal mechanisms of the various parameters are not linear.

The purpose of an orthogonal experimental design is to use the minority experiment to determine the law of multiple factors in order to obtain the best combination of programs. ***Eq***. 1 in the appendix can be widely used to meet the multifactor superposition for the orthogonal experiment. In combination with the previous result^19^, the influence of many factors is basically a linear superposition of each half-life increment. Therefore, the foam half-life and the impact of multifactor law function are roughly:

1 $${\rm{\Delta }}{T}_{1/2}(v,n,T,V)={k}_{v}{\rm{\Delta }}{T}_{ump}(v)+{k}_{n}{\rm{\Delta }}{T}_{urf}(n)+{k}_{T}{\rm{\Delta }}{T}_{mb}(T)+{k}_{V}{\rm{\Delta }}{T}_{yr}(V)$$

where *T*_*1/2*_ is the foam half-life. *T*_*ump*_(*v*) is a function regarding pump speed (*v*), which characterizes the half-life prepared in different *v* (Fig. 3b). *T*_*urf*_(*n*) is a function regarding surfactant concentration (*n*), while *T*_*mb*_(*T*) is a function regarding preparation temperature (*T*), and *T*_*yr*_(*V*) is a function regarding syringe size (*V*). *k*_*v*_, *k*_*n*_, *k*_*T*_, and *k*_*V*_ are overlay coefficients. According to the results of the multivariate experiments, the *T*_*ump*_(*v*), *T*_*urf*_(*n*), *T*_*mb*_(*T*), and *T*_*yr*_(*V*) functions have maximum values. Therefore, this results in a maximum value for Δ*T*_*1/2*_. By taking into account the value of *k*_*ci*_, the final half-life is estimated. For parameters that satisfy *Eq*. *1*, the *k*_*ci*_ values are approximately 1. On the contrary, it is less than 1. For more cases, more experimental verification is needed to determine the K value. This will provide the basis for the development of foam in various fields.

The time taken for the blood vessels to fibrose is between a few minutes and ten minutes. The current half-life of sclerosing foam is less than two minutes. Before vascular fibrosis, we should try to ensure that the foam is stable, not diluted, and does not flow to other parts. Therefore, it is still necessary to increase the stability of the foam as much as possible. We can greatly improve the half-life of the foam through comprehensive consideration of multiple factors. This result is desirable in a clinical scenario. However, the influence of other factors in this experiment was not considered, and their influencing parameters must be further explored. More research is warranted to determine the influence of various factors.

## Conclusions

Foam stability is a key issue in foam sclerotherapy, which is not only associated with patient safety, but also with potential improvements in foam sclerotherapy. In this study, the best pump speed was 250 mm/s for a 2.5 mL syringe. Four factors (syringe size, surfactant, preparation temperature, and pump speed) promoted markedly observable foam half-life, with the surfactant being the most influential, and syringe size having the least influence. The best combination to achieve a stable foam was a syringe size of 2.5 mL, addition of an appropriate surfactant, a preparation temperature of 10 °C, and an injection rate of 250 mm/s. This study determined that it is appropriate to consider using a combination of multiple factors to prepare a more stable foam and achieve favorable conditions, thereby avoiding unfavorable conditions in clinical settings. A half-life superposition formula was obtained which can guide subsequent foam stability studies. However, the superposition principle proposed in this work is expected to be the guiding theory for evaluating the influence of more factors on foam stability, which is also applied in other fields of engineering.

## Electronic supplementary material

Supplementary Information

## Data Availability

All data generated or analyzed during this study are included in this published article.

## References

[1] Ríos E, Sierralta A, Abarzúa M, Bastías J (2012). Esophageal and gastric varices. Rev Med Chil.

[2] Figueiredo LM, Trindade SC, Sarmento VA, Muniz WR, Valente RO (2012). Extensive gingival hemangioma in a 10-year-old boy treated by sclerotherapy: a case report. Journal of Oral & Maxillofacial Surgery Official Journal of the American Association of Oral & Maxillofacial Surgeons.

[3] Bergan JJ, Cheng VL (2008). Foam sclerotherapy: a textbook. Journal of Vascular & Interventional Radiology.

[4] Gillet JL (2016). Sclerotherapy is a safe method of treatment of chronic venous disorders in older patients: a prospective and comparative study of consecutive patients. Phlebology.

[5] Bergan J, Cheng V (2007). Foam sclerotherapy for the treatment of varicose veins. Vascular.

[6] Jia X (2007). Systematic review of foam sclerotherapy for varicose veins. BMJ.

[7] Weitz-Tuoretmaa A (2014). Efficacy of ok-432 sclerotherapy in treatment of lymphatic malformations: long-term follow-up results. Eur Arch Otorhinolaryngol.

[8] Forlee Martin V., Grouden Maria, Moore Dermot J., Shanik Gregor (2006). Stroke after varicose vein foam injection sclerotherapy. Journal of Vascular Surgery.

[9] Rao J, Goldman MP (2005). Stability of foam in sclerotherapy: differences between sodium tetradecyl sulfate and polidocanol and the type of connector used in the double-syringe system technique. Dermatologic Surg.

[10] Frullini A (2011). Commentary: An investigation into the influence of various gases and concentrations of sclerosants on foam stability. Dermatol Surg.

[11] Rial R (2014). Polidocanol foam stability in terms of its association with glycerin. Phlebology.

[12] Whiteley MS, Patel SB (2015). Modified tessari tourbillon technique for making foam sclerotherapy with silicone-free syringes. Phlebology, 30(9), 614. Modified Tessari Tourbillon technique for making foam sclerotherapy with silicone-free syringes. Phlebology.

[13] Bai T, Jiang W, Zhao W, Wan H, Fan Y (2016). Experimental study on influence of driving speed on foam stability in sclerotherapy for the treatment of varicose veins. J Biomed Eng.

[14] Bai T, Jiang W, Fan Y (2018). Influence of syringe volume on foam stability in sclerotherapy for varicose vein treatment. Dermatol Surg.

[15] Bai T (2018). Studies on foam decay trend and influence of temperature jump on foam stability in sclerotherapy. Vasc & Endovasc Surg.

[16] Nastasa V (2015). International Journal of Pharmaceutics.

[17] Wan H, Bai T, Jiang W, Huang X, Zhao W (2016). Development of automatic preparation device of sclerosing foam based on Tessari method. J Biomed Eng.

[18] Ramsay W, Shields J (1983). The Variation of Molecular Surface-Energy with Temperature. Philosophical Transactions of the Royal Society of London A.

[19] Bai, T. *et al*. A comparison of different surfactants on foam stability in foam sclerotherapy *in vitro*. *J Vasc Surg ahead of print*, 10.1016/j.jvs.2018.02.033 (2018). 10.1016/j.jvs.2018.02.033 29954633

