# Supplementary material for: Effect of Multiple Factors on Foam Stability in Foam Sclerotherapy
Source: Sci Rep. 2018 Oct 24;8:15683. doi: 10.1038/s41598-018-33992-w (PMC6200734; doi:10.1038/s41598-018-33992-w)
Supplement: Supplementary file 1 — Supplementary Information [file 41598_2018_33992_MOESM1_ESM.doc]

**Effect of Multiple Factors on Foam Stability in Foam Sclerotherapy**Taoping Bai ^1^, Wentao Jiang ^1, *^, Yu Chen ^1^, Fei Yan^1, 2, 3^, Zhi Xu^1, 2, 3^, and Yubo Fan ^4^

*^1^Laboratory of Biomechanical Engineering, Department of Applied Mechanics, Sichuan University, Chengdu 610065, China*

*^2^Department of Biomedical Engineering, The Hong Kong Polytechnic University, Hong Kong, China*

*^3^Institute for Disaster Management and Reconstruction (IDMR), Sichuan University — The Hong Kong Polytechnic University,*

*Chengdu 610065, China*

*^4^School of Biological Science and Medical Engineering, Beihang University, Beijing 100191, China*

**Word count:**2919

**Running Head**: Multiple Factors on for Foam Stability

**Corresponding Author:**

Wentao Jiang, Professor, Department of Applied Mechanics, Sichuan University, NanYihuan Road No. 24, WuHou District, Chengdu 610065, China. Tel: +86-13678134379; Fax: +86-028-85405140; E-mail: scubme@aliyun.com

**Appendix**

**Determination of linear correlation between parameters**

Whether there is any interaction between the parameters needs further verification and judgment. Therefore, if we consider all the situations, the equations to be determined are shown in Table 2. According to Table 2, it can estimate whether the foam half-life is linear superposition under the condition of multiple factors. First of all, different parameters are randomly numbered i, ranging from 1 to n. $t_{c_{i}}$ represents the value when the parameter is No. ***i***. $t_{0}$ is the value under the influence of no relevant parameters. $t_{c_{1}\sim c_{i}}$ represents the value of the mutual effect when the number of parameters is ***i***. $C_{n}^{m}$ represents the number of verification equations where ***m*** parameters work together. The total number of decision equations is $\mathop{\Sigma}\limits_{m=1}^{n} C_{n}^{m}$ for verification correlation between the n parameters.

Of course, based on the above ideas, we may be able to expand the definition. This may give us more inspiration and application. Then we define the generalized linear dependence determination equation (Table 4). Where $k_{c_{i}}$ is the superposition coefficient in the presence of the i-th parameter. This coefficient does not change with the size of the parameter. The linear superposition can also be made when a certain superposition coefficient $k_{c_{i}}$ is added. Then we can say that the influence of these parameters also satisfies the principle of linear superposition. Furthermore, the combined result of multiple factors is:

$\Delta t_{c_{1}\sim c_{n}}=\mathop{\Sigma}\limits_{i=1}^{n} k_{c_{i}}\Delta t_{c_{i}}$ （**Eq.**1）

**Table 4 Generalized multi-factor linear correlation determination equation**

| The number of correlation parameters to be verified | Equation to verify parameter dependencies | The number of equations |
| --- | --- | --- |
| 2 | $k_{c_{1}}(t_{c_{1}}-t_{0})+k_{c_{2}}(t_{c_{2}}-t_{0})=t_{c_{1},c_{2}}-t_{0}$ | $C_{n}^{2}$ |
| 3 | $k_{c_{1}}(t_{c_{1}}-t_{0})+k_{c_{2}}(t_{c_{2}}-t_{0})+k_{c_{3}}(t_{c_{3}}-t_{0})=t_{c_{1},c_{2},c_{3}}-t_{0}$ | $C_{n}^{3}$ |
| … | … | … |
| n | $\mathop{\Sigma}\limits_{i=1}^{n} k_{c_{i}}(t_{c_{i}}-t_{0})=t_{c_{1}\sim c_{n}}-t_{0}$ | $C_{n}^{n}$ |
